# Prognostic value of combined psoas muscle mass and controlling nutritional status in patients with pancreatic ductal adenocarcinoma: a retrospective cohort study

**DOI:** 10.1186/s12893-024-02395-2

**Published:** 2024-04-20

**Authors:** Shota Kuwabara, Yuta Takeuchi, Osamu Sato, Tomoko Mizota, Masaomi Ichinokawa, Katsuhiko Murakawa, Yuma Aoki, Keita Ishido, Koichi Ono, Satoshi Hirano

**Affiliations:** 1 Department of Surgery, Obihiro Kosei General Hospital, 1 West 14 South 10, Obihiro, 080-0024 Hokkaido Japan; 2 Department of Surgery, Asahikawa Red Cross Hospital, 1-1 Akebono 1-jo 1-chome, Asahikawa, 070-8530 Hokkaido Japan; 3 Department of Gastrointestinal Surgery II, Hokkaido University Faculty of Medicine, Kita 15-jo Nishi 7-chome, Kita-ku, Asahikawa, 070-8530 Hokkaido Japan

**Keywords:** Controlling nutritional status score, Pancreatic ductal adenocarcinoma, Prognosis, Psoas muscle mass index, Surgery

## Abstract

**Background:**

Pancreatic ductal carcinoma (PDAC) is an extremely poor prognostic disease. Even though multidisciplinary treatment for PDAC has developed, supportive therapies, such as nutritional therapy or perioperative rehabilitation to sustain and complete aggressive treatment, have not yet been well-established in PDAC. The aim of this study was to elucidate the relationship between the combined index using psoas muscle mass index (PMI) values and controlling nutritional status (CONUT) score and prognosis.

**Methods:**

We included 101 patients diagnosed with PDAC who underwent radical pancreatectomy with regional lymphadenectomy. The cut-off value was set at the first quartile (male, 6.3 cm^2^/m^2^; female 4.4 cm^2^/m^2^), and patients were classified into high PMI and low PMI groups. A CONUT score of 0 to 1 was classified as the normal nutritional status group, and 2 or more points as the malnutritional status group. Patients were further divided into three groups: high PMI and normal nutrition (good general condition group), low PMI and low nutrition (poor general condition group), and none of the above (moderate general condition group). We performed a prognostic analysis of overall survival (OS), stratified according to PMI values and CONUT scores.

**Results:**

In the poor general condition group, the proportion of elderly people over 70 years of age was significantly higher than that in the other groups (*p* < 0.001). The poor general condition group had a significantly worse prognosis than the good and moderate general condition groups (*p* = 0.012 and *p* = 0.037). The 5-year survival rates were 10.9%, 22.3%, and 36.1% in the poor, moderate, and good general condition groups, respectively. In multivariate analysis, poor general condition, with both low PMI and malnutrition status, was an independent poor prognostic factor for postoperative OS (hazard ratio 2.161, *p* = 0.031).

**Conclusions:**

The combination of PMI and CONUT scores may be useful for predicting the prognosis of patients with PDAC after radical surgery.

## Background

Pancreatic ductal carcinoma (PDAC) is the fourth leading cause of cancer-related deaths worldwide [1]. The 5-year survival rate is approximately 6–10% for all patients with PDAC, and even after radical resection, it is approximately 20%, making it an extremely poor prognostic disease [2]. Although multidisciplinary treatment for PDAC, including advanced surgical techniques, adjuvant chemotherapy, and neoadjuvant therapy, has made great strides in improving prognosis [3], supportive therapies, such as nutritional therapy or perioperative rehabilitation to sustain and complete aggressive treatment, have not yet been well-established.

Clinical outcomes of PDAC not only depend on tumor biology and treatment response but are also strongly influenced by the nutrition and performance status of the patients [4]. Body composition is considered important for predicting survival outcomes. Skeletal muscle wasting (i.e., sarcopenia) contributes to poor prognosis in patients with various cancers, such as colorectal, esophageal, and prostate cancer [5–7]. Additionally, a meta-analysis reported that sarcopenia is found in 38.7% of patients with PDAC and is an independent prognostic factor in multivariate analysis [8]. The early recognition and objective assessment of nutritional problems are key for PDAC management [9]. The Controlling Nutritional Status (CONUT) score as a tool to evaluate patient nutritional status has been used to predict the prognosis of patients with PDAC [10] and is an effective prognostic factor in patients with PDAC [11].

Although many studies have examined the association between sarcopenia or CONUT score and prognosis, few have examined the relationship between sarcopenia and CONUT score and prognosis. Thus, the aim of this study was to investigate the association between an index defined as combined sarcopenia (specifically, psoas muscle mass index [PMI]) and CONUT score and prognosis in patients with PDAC.

## Methods

### Patients

We retrospectively analyzed the medical records of 101 patients diagnosed with pancreatic cancer who underwent radical pancreatectomy with regional lymphadenectomy at the Department of Surgery, Obihiro Kosei General Hospital, between January 2009 and April 2021. Patients with intraductal papillary mucinous adenocarcinoma, those who underwent only exploratory laparotomy due to peritoneal dissemination, and those who underwent palliative surgery such as gastrointestinal bypass surgery or choledochojejunostomy were excluded from this study. Clinicopathological data were collected from patient records. All tumors were staged according to the 8th TNM classification system of the Union for International Cancer Control [12]. The present study was approved by the Institutional Review Board of Obihiro Kosei General Hospital (authorization number: 2021-067), and was carried out in compliance with the Helsinki Declaration. The opt-out recruitment method was applied to all patients, with providing an opportunity to decline to take part in the study. Informed consent was obtained from all the patients.

### Measurement and assessment of psoas muscle mass index

We measured the cross-sectional areas of the bilateral psoas muscles at the umbilical level using preoperative computed tomography (CT) images by the manual tracing method, and PMI was calculated as previously reported [13]. Cut-off values for PMI were established using the quartile method.

### Assessment of nutritional status

To assess preoperative nutritional status, we used the CONUT score, calculated from serum albumin, total lymphocyte count, and total cholesterol [14]. In the present study, we defined CONUT score > 2 as the “malnutritional” group and CONUT score < 2 as the “normal” group (Table 1).

**Table 1:** Assessment of nutritional status using CONUT scoring system

Albumin (g/dL)	≧3.5	3.49-3	2.99-2.5	2.5 >
	0	2	4	6
Total lymphocyte count (/µL)	≧1600	1599-1200	1199-800	800 >
	0	1	2	3
Total cholesterol (mg/dL)	≧180	179-140	139-100	100 >
	0	1	2	3
CONUT score	0-1	2-4	5-8	9-12
Nutritional status	normal	light	moderate	severe
Assessment	“normal”	“malnutrition”		

### Statistical analysis

Differences between the groups were analyzed using the Mann-Whitney U-test. Categorical variables were compared using Fisher’s exact test or χ^2^ -test. OS was defined as the time from surgery to death due to any cause. The proportion of OS was calculated using the Kaplan-Meier method. Comparisons between groups were performed using the log-rank test. Univariate and multivariate analyses were performed using a Cox proportional hazards model. Differences were considered statistically significant at *p* < 0.05 [15]. Statistical analyses were performed using JMP Pro 17 (SAS Institute Inc., Cary, NC, USA).

## Results

### Comparison of PMI values by sex

**Fig. 1:**
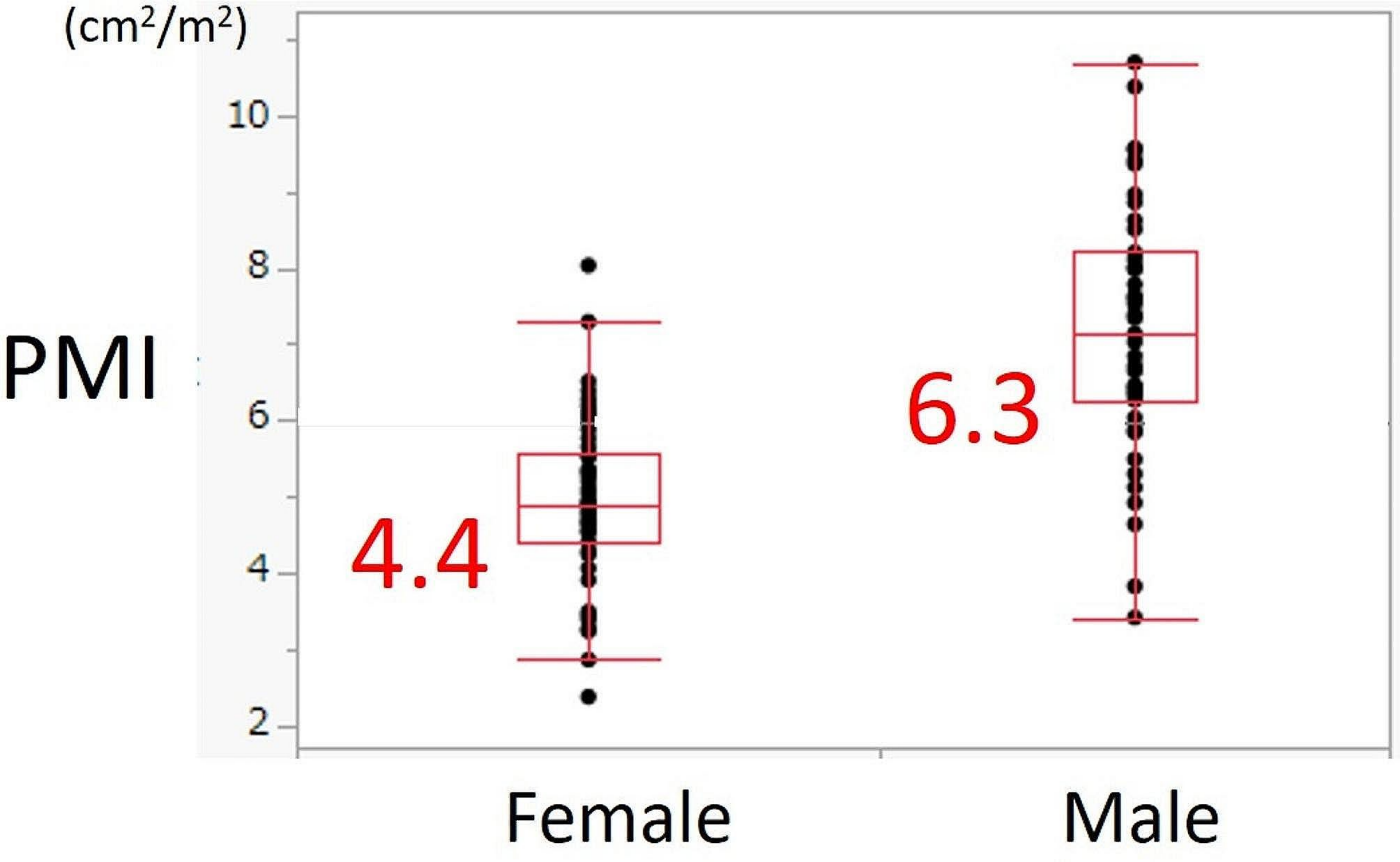
PMI values by sex. Significant differences between male and female patients were found (*p* < 0.001). The first quartile (6.3 cm^2^/m^2^ for male and 4.4 cm^2^/m^2^ for female) was set as the cut-off value

The median PMI was significantly higher for male patients than for female patients (7.2 cm^2^/m^2^ [range, 3.4–10.7 cm^2^/m^2^] and 4.9 cm^2^/m^2^ [range, 2.3-8.0 cm^2^/m^2^], respectively; *p* < 0.001). The first quartile, 6.3 cm^2^/m^2^ for male and 4.4 cm^2^/m^2^ for female patients, was set as the cut-off value (Fig. 1). The patients were then classified into high and low PMI groups based on the cut-off values.

### Evaluation of nutritional status according to CONUT score

According to the assessment of nutritional status using the CONUT score, 47 patients (46.5%) were classified into the “normal” group and 54 patients (53.5%) were classified into the “malnutrition” group.

### Prognostic analysis

We performed a prognostic analysis of OS, stratified according to PMI values and CONUT scores. The high PMI group had better prognosis than the low PMI group; however, the difference was not significant (*p* = 0.1160). Similarly, the normal nutritional status group had a better prognosis than the malnutrition status group, but the difference was not significant (*p* = 0.1129) (Fig. 2).

**Fig. 2A:**
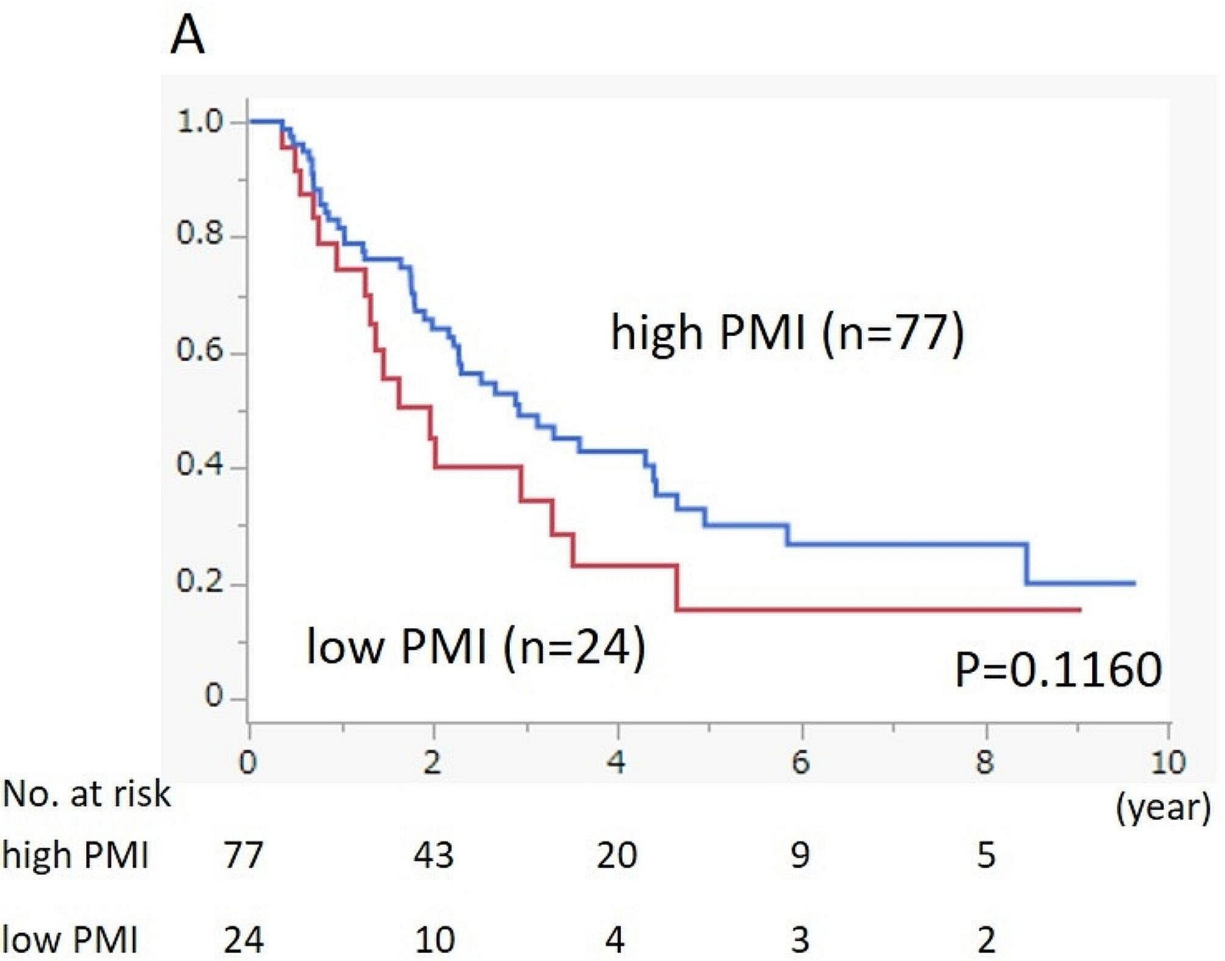
Overall survival of the 101 patients with PDAC according to the PMI values

**Fig. 2B:**
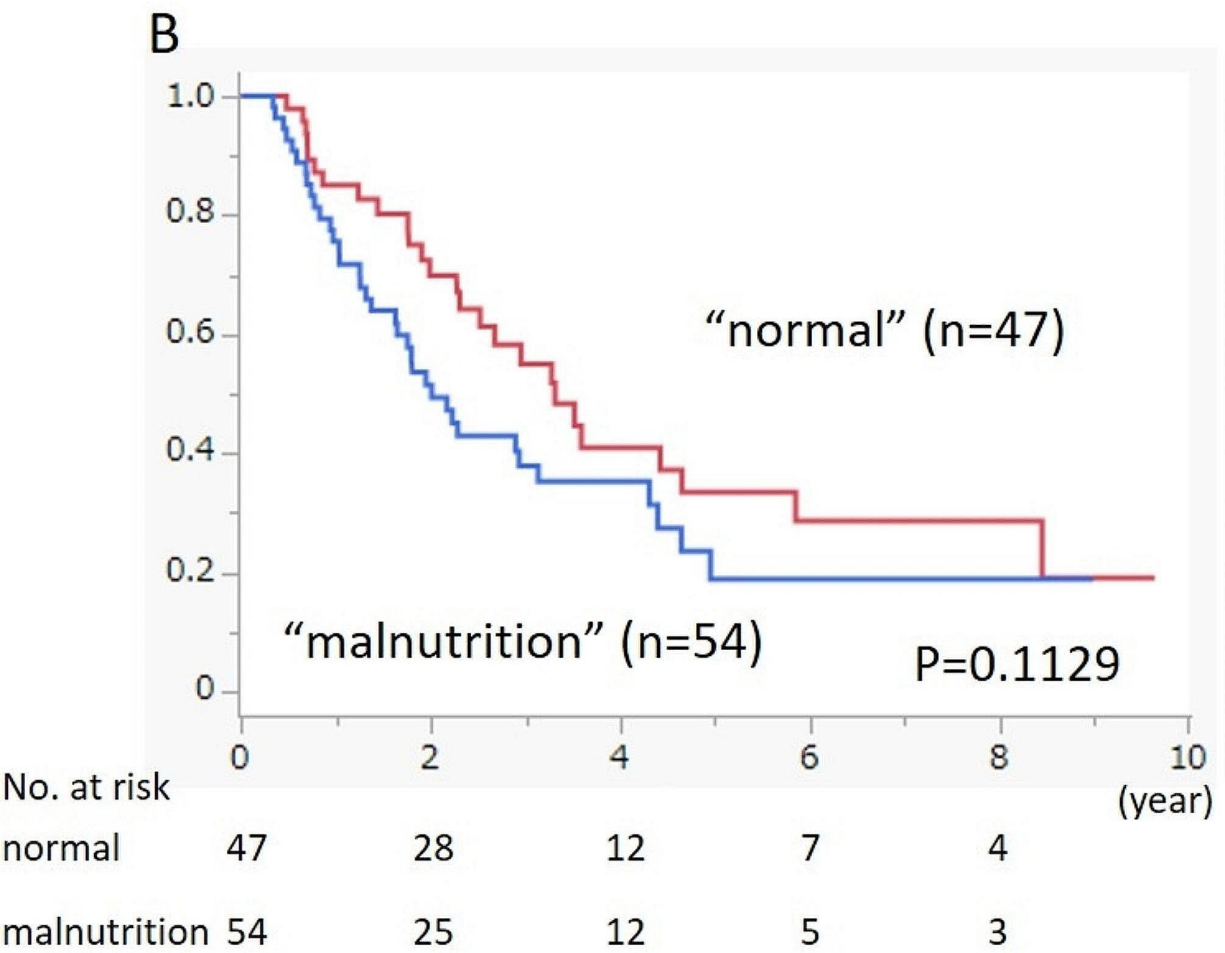
Overall survival of the 101 patients with PDAC according to CONUT score. The OS curves were estimated using the Kaplan-Meier method, and differences between the groups were evaluated using a log-rank test. The number of patients at risk is shown under the OS curves

Based on these results, we stratified the patients into three groups based on the combined PMI values and nutritional status based on the CONUT score. Patients with both low PMI and in the malnutritional group were defined as in “poor” general condition (*n* = 16), those with high PMI or in the normal nutritional group (but not both) were defined as in “moderate” general condition (*n* = 46), and those with both high PMI and normal nutritional status were defined as in “good” general condition (*n* = 39). The relationships between the clinicopathological characteristics and each of the three groups are summarized in Table 2. In the poor general condition group, the proportion of elderly people over 70 years of age was significantly higher than that in the other groups (*p* < 0.001). There were no significant differences in sex; tumor location; presence or absence of portal vein resection; pathological T, N, and M factors with or without adjuvant chemotherapy; or postoperative complications.

**Table 2:** Clinicopathological background characteristics of the patients

*N*=101		poor (*n*=16)	moderate (*n*=46)	good (*n*=39)	p-value
Age	≧70	14	34	16	<0.001
	70 >	2	12	23	
Sex	Male	9	19	19	0.552
	Female	7	27	20	
Location	Ph	9	27	24	0.928
	Pbt	7	19	15	
PVR	with	5	10	5	0.269
	without	11	36	34	
pT	0/is/1	2	14	14	0.224
	2/3	14	32	25	
pN	0	8	17	13	0.507
	1/2	8	29	26	
pM	0	0	0	0	0.295
	1	0	2	0	
Adjuvant	+	11	29	29	0.535
	-	5	17	10	
Complication	+	8	26	19	0.755
	-	8	20	20	

Ph: pancreatic head; Pbt: pancreatic body or tail; PVR: portal vein resection

**Fig. 3:**
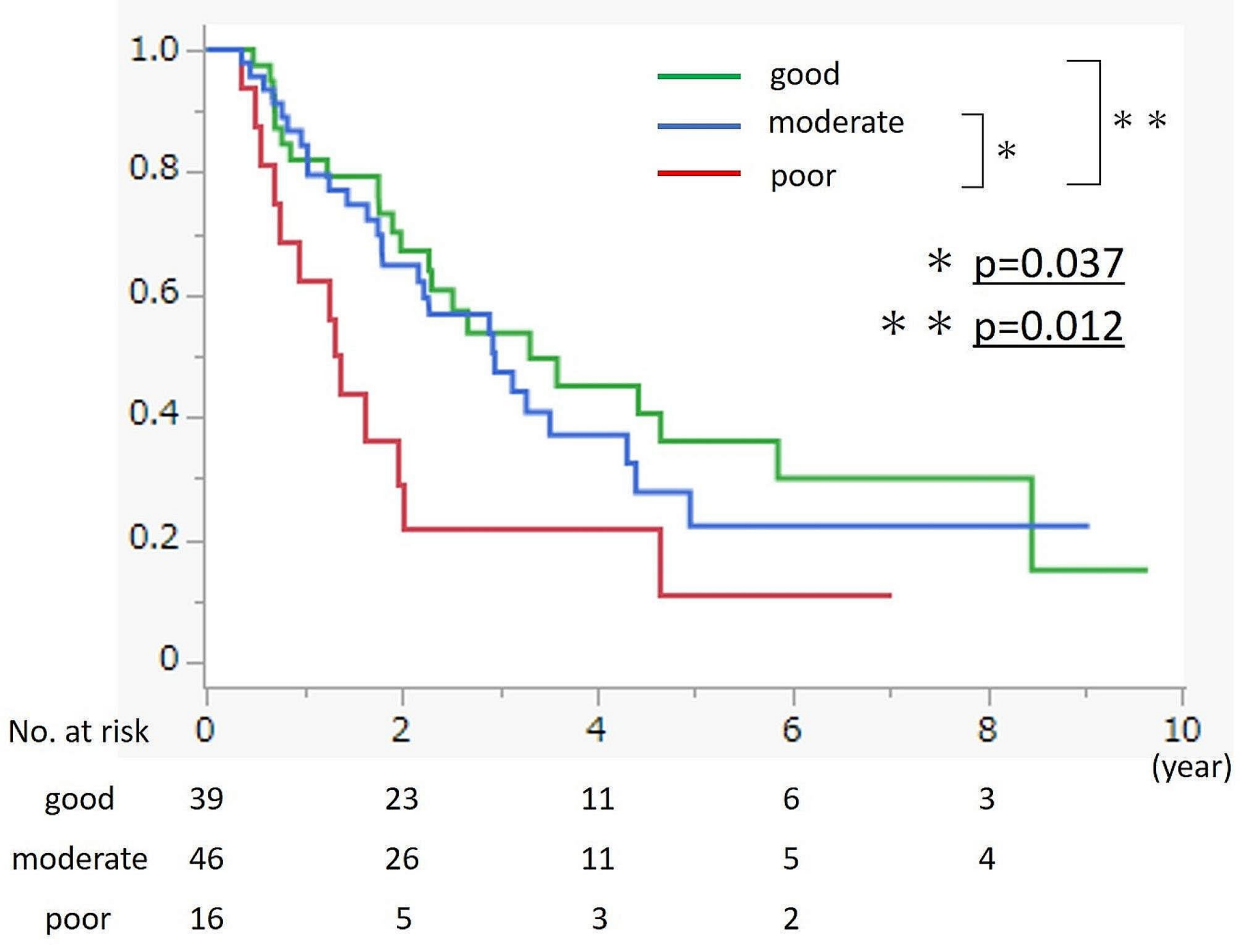
Survival rates in patients with PDAC according to the combination of PMI values and CONUT score. The OS curves were estimated using the Kaplan-Meier method, and differences among the three groups were evaluated using a log-rank test. The number of patients at risk is shown under the OS curves

The median follow-up period was 733 days. Figure 3 shows the Kaplan-Meier curves for the patients’ general condition based on the combination of PMI values and CONUT scores. The poor general condition group had a significantly worse prognosis than the moderate general condition group (*p* = 0.037) and, thus, the good general condition group (*p* = 0.012). The 5-year survival rates were 10.9%, 22.3%, and 36.1% in the poor, moderate, and good general condition groups, respectively. Univariate and multivariate analyses were performed by adding the patients’ general condition to the clinicopathological factors. In multivariate analysis, poor general condition (hazard ratio [HR]: 2.161, 95% confidence interval [CI]: 1.071–4.359, *p* = 0.031), with both low PMI and malnutrition status, was determined to be an independent prognostic factor of a poor outcome (Table 3).

**Table 3:** Univariate and multivariate analyses of OS of the clinicopathological factors and the patients’ general condition

*N*=101				Univariate analysis				Multivariate analysis		
Variables				HR	95%CI	*p*-value		HR	95%CI	*p*-value
General condition	poor	/ good		2.390	(1.197-4.772)	0.014		2.161	(1.071-4.359)	0.031
	poor	/ moderate		2.065	(1.060-4.024)	0.033				
	good	/ moderate		0.863	(0.491-1.520)	0.612				
Age	≧70	/ <70		1.340	(0.784-2.289)	0.284				
Sex	Male	/ Female		1.305	(0.792-2.150)	0.297				
Location	Ph	/ Pbt		0.768	(0.461-1.280)	0.311				
Tumor size	>2 cm	/ 2 cm≧		1.993	(1.068-3.718)	0.030		1.944	(0.952-3.971)	0.068
Nodal metastasis	+	/ -		1.884	(1.078-3.296)	0.026		1.469	(0.794-2.718)	0.221
Adjuvant	+	/ -		0.659	(0.393-1.106)	0.115				
Complication	+	/ -		1.375	(0.829-2.280)	0.218				

OS: overall survival; HR: hazard ratio; CI: confidence interval; Ph: pancreatic head; Pbt: pancreatic body or tail

## Discussion

The purpose of the present study was to investigate the relationship between the combined index, according to the PMI and CONUT scores, and prognosis in patients with PDAC. We demonstrated that patients with a poor general condition, defined as both decreased skeletal muscle (i.e., low PMI) and malnutrition (i.e., CONUT score > 2), had a significantly worse prognosis than other patients, and this was found to be an independent unfavorable prognostic factor in multivariate analysis.

Sarcopenia is defined as a syndrome characterized by progressive and generalized loss of skeletal muscle mass and strength, with a risk of adverse outcomes such as physical disability, poor quality of life, and death. It is recommended that the definition of sarcopenia should include not only low muscle mass but also low muscle function [16]; however, most previous studies have investigated only skeletal muscle mass to define sarcopenia. Because we investigated only PMI in this study, we did not use the term sarcopenia but instead expressed it as decreased skeletal muscle. Based on the recent consensus of the Asian Working Group for Sarcopenia, CT imaging at the level of the third lumbar vertebra is an effective imaging modality for the clinical detection of sarcopenia [17], but we measured the cross-sectional area of the bilateral psoas muscles at the umbilical level using preoperative CT imaging and calculated the PMI following a previous report [13]. Interestingly, the cut-off values of the PMI obtained from this study (male, 6.3 cm^2^/m^2^; female, 4.4 cm^2^/m^2^) were approximately the same as those used in the sarcopenia criteria proposed by the Japan Society of Hepatology (male, 6.36 cm^2^/m^2^; female, 3.92 cm^2^/m^2^) [18]. Several studies have shown that the low PMI group has a significantly worse prognosis and is an independent prognostic factor after curative surgery, previously [13, 19]. Measurement of the psoas muscle area is a simple and convenient method, and PMI values may be useful for predicting the postoperative prognosis of patients with PDAC.

Malnutrition is prevalent in patients with cancer and can affect short- and long-term outcomes. Nutritional status has a prognostic capacity for post-treatment long-term outcomes, including disease progression and survival [20]. The CONUT score is a newly proposed index for objectively assessing patients’ nutritional status [14], which has prognostic value with respect to poor survival in patients with cancer [9–11, 21]. The CONUT score was calculated using serum albumin, total lymphocyte count, and total cholesterol values. The predictive effect of the CONUT score can be attributed to several factors. First, serum albumin has long been regarded as an important marker of nutritional status, and low albumin levels are associated with advanced cachexia and perioperative outcomes [22]. Second, total lymphocyte count is an immunological indicator. The decrease in lymphocyte and primary T lymphocyte levels indicates an inadequate immune response against a tumor [23]. Third, cholesterol is essential for the maintenance of cell membrane function, which is crucial for signal transduction. Decreased cholesterol levels can affect the antitumor activity of immunocompetent cells [24]. These findings suggest that the CONUT score has higher prognostic accuracy and is superior in predicting survival in different types of cancer [21].

Despite the availability of the PMI or CONUT score reported previously, this study did not find the PMI or CONUT score alone to be effective in predicting prognosis. This may have been owing to the small sample size. Therefore, we devised a new evaluation method, a combination of PMI values and CONUT scores, and proposed it as a prognostic indicator for the first time. We revealed that patients with poor general condition, defined as both low PMI and malnutrition, expressed as a CONUT score > 2, had significantly worse prognosis, and was found to be an independent prognostic indicator in PDAC following radical surgery. A combined assessment of decreased skeletal muscle mass and nutritional impairment may accurately reflect a patient’s general condition. Indeed, in patients with PDAC, poor oral nutritional intake, catabolism due to malignancy, and reduced intestinal absorption due to obstruction can synergistically affect the nutritional status and lead to malnutrition and loss of muscle mass [25]. Pancreatic exocrine insufficiency also contributes to malnutrition and weight loss. Pancreatic enzymes are essential for the degradation and absorption of fat and liposoluble vitamins; thus, pancreatic enzyme deficiency results in steatorrhea and severe maldigestion [26]. As respects the relationship between a combined index of skeletal muscle mass and malnutrition and patients’ prognosis, there was another report that the combination of the geriatric nutritional risk index (GNRI) and psoas muscle volume (PMV) might be useful to predict prognosis in older patients with pancreatic cancer. Previously, it has demonstrated that patients with low GNRI and low PMV had the worst pancreatic cancer prognosis [27]. Our findings strongly suggest that poor nutritional status and decreased skeletal muscle mass negatively affect long-term clinical outcomes after pancreatic surgery. Prehabilitation regimens based on exercise such as aerobic and resistant activity and nutritional support focused on maximizing energy and protein intake should be required for these patients to improve their prognosis.

This study has several limitations. First, this was a single-institute, retrospective cohort study with a small population of Asian patients. This might have resulted in a selection bias that limited the generalizability of our findings to patients worldwide. Second, the PMI values of each patient were measured using a manual tracing method, which led to measurement errors by the examiner. To avoid measurement errors, the use of more objective instruments such as bioelectrical impedance analysis or dual-energy X-ray absorptiometry to measure skeletal muscle mass is necessary. Third, the cut-off value of PMI obtained from this study was originally defined using the quartile method without consensus. For universalization, cut-off values based on international standards should be used. Numerous multi-institutional prospective studies involving various ethnicities are necessary to confirm our findings.

## Conclusions

In conclusion, we described, for the first time, the usefulness of the combination of the PMI and CONUT scores in predicting the prognosis of patients with PDAC after radical surgery. Preoperative management, including rehabilitation and nutritional support, might impact patients with a poor general condition and contribute to improving their prognosis.

## Data Availability

The datasets used and/or analysis during the current study are available from the corresponding author on reasonable request.
